# An improved synergistic dual-layer feature selection algorithm with two type classifier for efficient intrusion detection in IoT environment

**DOI:** 10.1038/s41598-025-91663-z

**Published:** 2025-03-07

**Authors:** G Logeswari, K Thangaramya, M Selvi, J. Deepika Roselind

**Affiliations:** 1 School of Computer Science and Engineering, Vellore Institute of Technology, Chennai, India; 2 School of Computer Science and Engineering, Vellore Institute of Technology, Vellore, India

**Keywords:** Intrusion detection system, Internet of things, Dynamic feature selection, Machine learning, Anomaly detection, Security systems, Engineering, Electrical and electronic engineering

## Abstract

In an era of increasing sophistication and frequency of cyber threats, securing Internet of Things (IoT) networks has become a paramount concern. IoT networks, with their diverse and interconnected devices, face unique security challenges that traditional methods often fail to address effectively. To tackle these challenges, an Intrusion Detection System (IDS) is specifically designed for IoT environments. This system integrates a multi-faceted approach to enhance security against emerging threats. The proposed IDS encompasses three critical subsystems: data pre-processing, feature selection and detection. The data pre-processing subsystem ensures high-quality data by addressing missing values, removing duplicates, applying one-hot encoding, and normalizing features using min-max scaling. A robust feature selection subsystem, employing Synergistic Dual-Layer Feature Selection (SDFC) algorithm, combines statistical methods, such as mutual information and variance thresholding, with advanced model-based techniques, including Support Vector Machine (SVM) with Recursive Feature Elimination (RFE) and Particle Swarm Optimization (PSO) are employed to identify the most relevant features. The classification subsystem employ two stage classifier namely LightGBM and XGBoost for efficient classification of the network traffic as normal or malicious. The proposed IDS is implemented in MATLAB by using TON-IoT dataset with various performance metrics. The experimental results demonstrate that the proposed SDFC method significantly enhances classifier performance, consistently achieving higher accuracy, precision, recall, and F1 scores compared to other existing methods.

## Introduction

The IoT has revolutionized the way to interact with technology by connecting a multitude of devices, ranging from everyday household appliances to sophisticated industrial systems. This interconnected network enables seamless communication, real-time monitoring, and automation, driving significant improvements in efficiency, convenience, and decision-making across various domains^1,2^. The rapid growth of IoT has introduced a range of security concerns that are distinct from those faced in traditional network environments. Numerous IoT devices are designed with minimal security features due to their resource constraints, such as limited processing power and battery life. The lack of efficient security mechanisms attracts the intruders who can able to exploit weakness to launch the different attacks which will compromise the security requirement of the system^3–5^. Additionally, the heterogeneity of IoT devices encompassing different manufacturers, communication protocols, and operating systems complicates the implementation of uniform security measures. Traditional security solutions, including firewalls and antivirus programs, are often inadequate for the dynamic nature of IoT networks. These solutions are typically optimized for more static environments and may struggle to handle the voluminous and heterogeneous data which are generated by devices of IoT^5^. As a result, there is a growing need for specialized IDS that can effectively monitor, detect, and respond to threats in real-time. IDS play a crucial role in safeguarding IoT networks by continuously monitoring network traffic, analyzing data patterns, and identifying potential security breaches. An effective IDS for IoT must be capable of handling large volumes of data generated by numerous devices and must be adaptable to the rapidly evolving threat landscape^6^. Traditional IDS solutions often fall short in this regard due to their inability to cope with the scale and diversity of IoT environments. Developing an IDS specifically designed to meet IoT requirements is crucial for accurately detecting and countering sophisticated attacks, ensuring the security and integrity of IoT networks^7–9^. Effective feature extraction provides important role in identification of intrusions.

Feature selection is one of the important factors for efficient identification for intrusion. Feature selection and machine learning plays integral role in enhancing the effectiveness of Intrusion Detection and Prevention System (IDPS) solutions. In IDS, feature selection process involves selection of the most important and relevant attributes from a dataset, which helps in reducing dimensionality, improving computational efficiency, and enhancing model performance. By focusing on significant features, feature selection techniques can reduce the noise and redundancy in the data, thereby improving the accuracy in intrusion detection. The feature selection algorithms, can continuously improve the classification algorithm performance for making efficient decision for the resource constraints and dynamic IoT environments^10^. Despite advancements in security technologies, existing IDS solutions fails to provide efficient solution for the dynamic IoT networks. The high-dimensional and heterogeneous nature of IoT data can overwhelm traditional detection systems, leading to reduced accuracy and increased in false positive rate. Furthermore, most of the existing solutions lack the adaptability required to handle evolving threats^11–15^. Hence, there is need to develop an IDS that integrates advanced feature selection techniques and machine learning (ML) models to overcome these limitations and provide a more effective and adaptive security solution for IoT networks. Motivated from the observation from these existing approaches, there is need to enhance the security of IoT networks amidst an increasingly sophisticated threat landscape. As IoT devices become more widespread, the potential impact of security breaches grows, affecting not only individual users but also critical infrastructure and public safety. Addressing the limitations of current IDS solutions through innovative feature selection and machine learning approaches is crucial for developing a more robust and resilient security framework. The main aim of this work is to provide efficient intrusion and reduce the false alarm in IoT environments. Table 1 provides the list of acronyms used in the proposed IDS.

The primary objectives of this research are:

1. To design and implement a cutting-edge IDS that leverages advanced feature selection techniques and ML algorithms tailored for IoT networks.
2. Assess the performance of the proposed IDS using the TON-IoT dataset, focusing on its ability to accurately detect and mitigate various types of cyber-attacks.
3. Conduct a comparative analysis of different machine learning models and feature selection methods to evaluate their impact on intrusion detection.

**Table 1:** Acronyms used in the proposed IDS.

Acronym	Full Form
**IDS**	Intrusion Detection System
**TON-IoT**	The Onion IoT (dataset)
**SDFC**	Synergistic Dual-Layer Feature Selection
**SITS**	Statistical and Information-Theoretic Selection
**MBOS**	Model-Based Optimization Selection
**SVM-RFE**	Support Vector Machine - Recursive Feature Elimination
**PSO**	Particle Swarm Optimization
**MI**	Mutual Information
**Variance Thresholding**	Method for feature selection based on variance
**Correlation Analysis**	Method for feature selection based on feature correlations
**LightGBM**	Light Gradient Boosting Machine
**XGBoost**	Extreme Gradient Boosting
**PDF**	Probability Density Function
**JPD**	Joint Probability Distribution
**RFE**	Recursive Feature Elimination
**CI**	Classification Instance
**PSO**	Particle Swarm Optimization
**SVM**	Support Vector Machine

## Literature survey

IoT networks are vulnerable to a wide range of cyber threats due to their heterogeneous and interconnected nature. The growing complexity of these networks necessitates advanced security solutions to protect against increasingly sophisticated attacks. Recent research has focused on developing robust IDS that can effectively address security challenges in IoT environment. Among them, Tsimenidis et al.^16^ have explored the application of deep learning techniques for IDS in IoT networks. They have highlighted the limitations of traditional intrusion detection methods in addressing the complex and dynamic nature of IoT environments. The authors have reviewed various deep learning models emphasizing their potential to improve detection accuracy and reduce false positives. Their paper also discussed the datasets used for evaluating these models, presented case studies demonstrating practical applications, and identified challenges such as scalability and data privacy. Maghrabi^17^ have addressed the need for advanced network IDS tailored to IoT environments. Their study focused on automating intrusion detection processes to enhance security in IoT networks, integrating machine learning algorithms to improve accuracy and efficiency. Moreover, author have proposed a framework that combined feature extraction, anomaly detection, and classification algorithms to identify threats in real-time. Their evaluation showed that automated methods significantly improved detection rates and reduced false positives compared to traditional approaches, effectively addressing the unique challenges of IoT networks. Gupta et al.^18^ have explored a hybrid approach combining optimization techniques and deep learning for enhancing IDS. They have integrated methods such as Particle swarm optimization (PSO) and genetic algorithms with deep learning models to improve feature selection and model training. Their study showed that this hybrid method significantly enhanced detection accuracy and reduced false positives compared to traditional approaches. Their system was evaluated using various datasets, demonstrating its effectiveness in addressing the challenges of detecting sophisticated intrusions.

Ullah et al.^19^ have proposed an IDS system for classifying Distributed Denial of Service (DDoS) attacks in IoT networks. They have introduced a dynamic approach which adjusts feature selection in real-time to enhance classification accuracy and reduce false positives. Their evaluation showed that this method significantly improved DDoS attack classification performance compared to static approaches. However, the study also acknowledged limitations, such as the potential computational overhead of real-time attribute selection and the need for extensive tuning to adapt the technique to various network conditions and attack types. These limitations could impact the scalability and practical deployment of the proposed method in large-scale IoT networks. Shakir and Mohsin^20^ have conducted a comparative analysis of IDS, focusing on algorithm classifications and feature selection techniques. Their study evaluated various intrusion detection algorithms and feature selection methods to identify their strengths and weaknesses. They have compared traditional techniques with advanced approaches, assessing their performance in terms of detection accuracy, false positives, and computational efficiency. The analysis highlighted that while advanced methods often offer improved performance, they may require more complex configurations and higher computational resources. Their study underscored the importance of selecting appropriate algorithms and feature selection techniques based on specific network requirements and attack scenarios. However, it also noted limitations, such as the challenge of generalizing results across different types of networks and the potential trade-offs between accuracy and computational overhead in practical implementations.

Maheswaran et al.^21^ have provided a critical review of IDS in IoT networks based on machine learning approaches. Their survey, analysed various machine learning techniques applied to IDS, including supervised, unsupervised, and hybrid methods. They have discussed the effectiveness of these approaches in detecting diverse types of intrusions, highlighting their strengths in terms of accuracy and adaptability. However, the review also pointed out several limitations, such as the high computational costs associated with some machine learning models, the challenge of handling large-scale IoT data, and the need for extensive training datasets to achieve robust performance. Natarajan et al.^22^ have conducted a survey on the effective utilization of ML algorithms in IoT-based IDS. Their study reviewed various ML algorithms applied to intrusion detection in IoT networks, focusing on their effectiveness in identifying and mitigating security threats. The authors assessed the performance of different algorithms, including supervised and unsupervised learning methods, and discussed their strengths in enhancing detection accuracy and adapting to evolving threats. However, the survey also highlighted limitations, such as the high computational resources required for some algorithms, challenges in handling the vast amount of data which are generated by IoT devices, and the need for tailored solutions to address specific network and attack characteristics.

Natarajan et al.^23^ have proposed an efficient feature selection algorithm to enhance ML-based IoT intrusion detection systems. Their proposed approach aimed to optimize feature selection, reducing computational complexity while maintaining high detection accuracy. Their method focused mainly on identifying the most relevant features, helping to minimize noise and irrelevant data, which in turn improved the efficiency of the machine learning models used for intrusion detection. While the results showed significant performance gains, the authors noted significant limitations such as the method’s dependency needed for further evaluation across different IoT environments. Balancing optimal performance with minimizing computational resources was highlighted as a challenge for real-world implementation. Mani et al.^24^ have proposed a novel IDPS using a hybrid deep neural network in cloud environments. Their system employs various hybrid deep learning models to provide efficient classification of various types of attacks in cloud-based networks. By integrating multiple neural network architectures, their approach aimed to improve accuracy and efficiency in identifying and mitigating security breaches. The authors demonstrated that their hybrid model outperformed traditional methods, particularly in handling dynamic patterns which detects complex attack. However, their work also highlighted challenges, such as the high computational demands of deep learning models and the need for efficient resource management to ensure scalability and real-time performance in large cloud networks.

Idrissi et al.^25^ have analyzed security requirements of IoT using deep learning models. Their review examined various deep learning techniques applied to securing IoT networks, highlighting the importance of these methods in mitigating intrusions. The authors analyzed a range of deep learning models, including Convolutional Neural Network (CNN), Recurrent Neural Network (RNN), and autoencoders, evaluating their performance in different IoT security scenarios. Their review revealed that deep learning-based IDS offered significant improvements in detection accuracy and adaptability over traditional methods. However, challenges such as the high computational cost of deep learning models, the need for large labeled datasets, and issues related to real-time processing. To overcome the challenges of high computational complexity, low detection accuracy, and elevated false positive rates, two innovative IDS approaches inspired by ensemble learning have been proposed^26^. The first, CNN-GWO-Voting, integrates CNN for feature extraction, Gray Wolf Optimization (GWO) for feature selection, and a soft voting mechanism to combine predictions from four base classifiers: random forest, SVM, decision tree, and XGBoost. Evaluated on the CIC-IoT-2023 dataset, this approach achieves exceptional performance with 99.15% accuracy, 0.99 precision, and 0.99 recall, while reducing computational complexity by selecting only 15 out of 46 features. The second approach utilizes a hybrid ensemble classifier that combines logistic regression, naïve bayes, SVM, K-Nearest Neighbors (KNN), and multilayer perceptron, with ensemble techniques such as voting, stacking, bagging, and boosting. Boosting yielded the highest performance, achieving 98.16% accuracy, 0.99 precision, and 0.98 recall on the same dataset. Both approaches significantly outperform existing methods, providing notable advancements in feature extraction, optimization, and ensemble strategies for enhancing cyber-physical systems (CPS) security.

A novel intrusion detection framework that integrated ensemble-based techniques for CPS was introduced^27^. This framework leveraged the strengths of multiple methods for intrusion detection and classification, potentially addressing the limitations of existing IDS solutions for CPS. A comprehensive overview of advanced intrusion detection techniques for CPSs was provided. The review explored various features of CPS, industrial protocols, and anomaly detection methods employed in intrusion detection. Additionally, a taxonomy of IDS for CPS and a classification of attacks and threats targeting CPS were proposed. Their review also highlighted the key research challenges in developing IDS for CPS. Recent studies on intrusion detection in Industrial IoT have highlighted the challenge of addressing sophisticated cyber threats while maintaining computational efficiency. Researchers have investigated the use of genetic algorithms (GA) for effective feature selection, which, when paired with Long Short-Term Memory (LSTM) networks, can improve anomaly detection accuracy. The incorporation of attention mechanisms has been shown to enhance model performance by focusing on crucial features within the data. The Adam optimizer, particularly its modified version (mADAM), has been explored for optimizing LSTM networks to achieve better detection results. Moreover, incorporating Explainable AI (XAI) techniques like Shapley Additive Explanations (SHAP) has become a focal point to improve the transparency and interpretability of threat detection models^28^. The research^29^ introduced a novel approach combining a Modified Genetic Algorithm (MGA) for feature selection and a GA for optimizing LSTM parameters within an Edge Computing (EC) framework. Their LSTM model’s parameters, including the number of hidden layers, were fine-tuned using a GA fitness function. In their system, MGA was customized to improve feature selection, enhancing resource efficiency in IoT devices and edge nodes. The approach aimed to maximize the LSTM’s effectiveness in detecting patterns in IoT network traffic through optimal hyperparameters, architecture, and training strategies. To address class imbalance, the focal loss function was employed to prioritize learning from minority classes, further improving model performance.

Their proposed model^30^ integrates Shapley Additive Explanations (SHAP) for transparency and uses hybrid bidirectional LSTM with autoencoders to reduce IoV network traffic dimensionality, improving efficiency. It employs Barnacle Mating Optimizer (BMO) to fine-tune hyperparameters of models like ResNet and MobileNet, enhancing detection without requiring large datasets. Experimental results showed perfect performance (100% accuracy, precision, recall, F1-score) in binary-class internal vehicular networks and 99.88% accuracy in multi-class external networks. Their model outperformed existing methods in detecting zero-day attacks and is scalable for real-time applications with limited computational resources. Their study^31^ introduced a hybrid Autoencoder and Modified Particle Swarm Optimization (HAEMPSO) for feature selection combined with deep neural network (DNN) classification. The model was evaluated on the UNSW-NB15 and BoT-IoT datasets, achieving 98.8% accuracy and 99.9% detection rate (DR) for Generic attacks in the UNSW-NB15 dataset, and 99.22% accuracy with a 97.79% DR for DDoS HTTP attacks in the BoT-IoT dataset. The proposed HAEMPSO-DNN model demonstrated superior performance in comparison to existing machine learning approaches, excelling in both accuracy and DR. The paper^32^ proposed an IDS utilizing an autoencoder for feature dimensionality reduction, trained on network flow data through a Deep Convolutional Neural Network (DCNN) and LSTM. The system was evaluated on the ICS dataset and gas pipeline data from Mississippi State University. The LSTM model achieved over 99% accuracy and an AUC-ROC of 99.50% for the ICS data, while the DCNN model achieved 96.0% accuracy and an AUC-ROC of 97.20% for the gas pipeline network, with minimal false positives and negatives. The results demonstrated that LSTM outperformed DCNN in anomaly detection for ICS. Both models were effective in time series prediction tasks, offering a cost-effective, unsupervised solution suitable for real-world deployment.

### Research gap

Securing IoT networks against increasingly sophisticated cyber threats demands advanced approaches that go beyond traditional methods. Deep learning and machine learning-based IDS have demonstrated significant potential in enhancing detection accuracy and reducing false positives in complex and dynamic IoT environments. The integration of feature selection techniques further optimizes these systems, balancing computational efficiency with performance. However, challenges such as high computational costs, scalability, and the need for large labelled datasets persist. Ongoing research is essential to address these limitations and to develop more robust, efficient, and scalable security solutions tailored for IoT networks.

## Proposed system

**Fig. 1:**
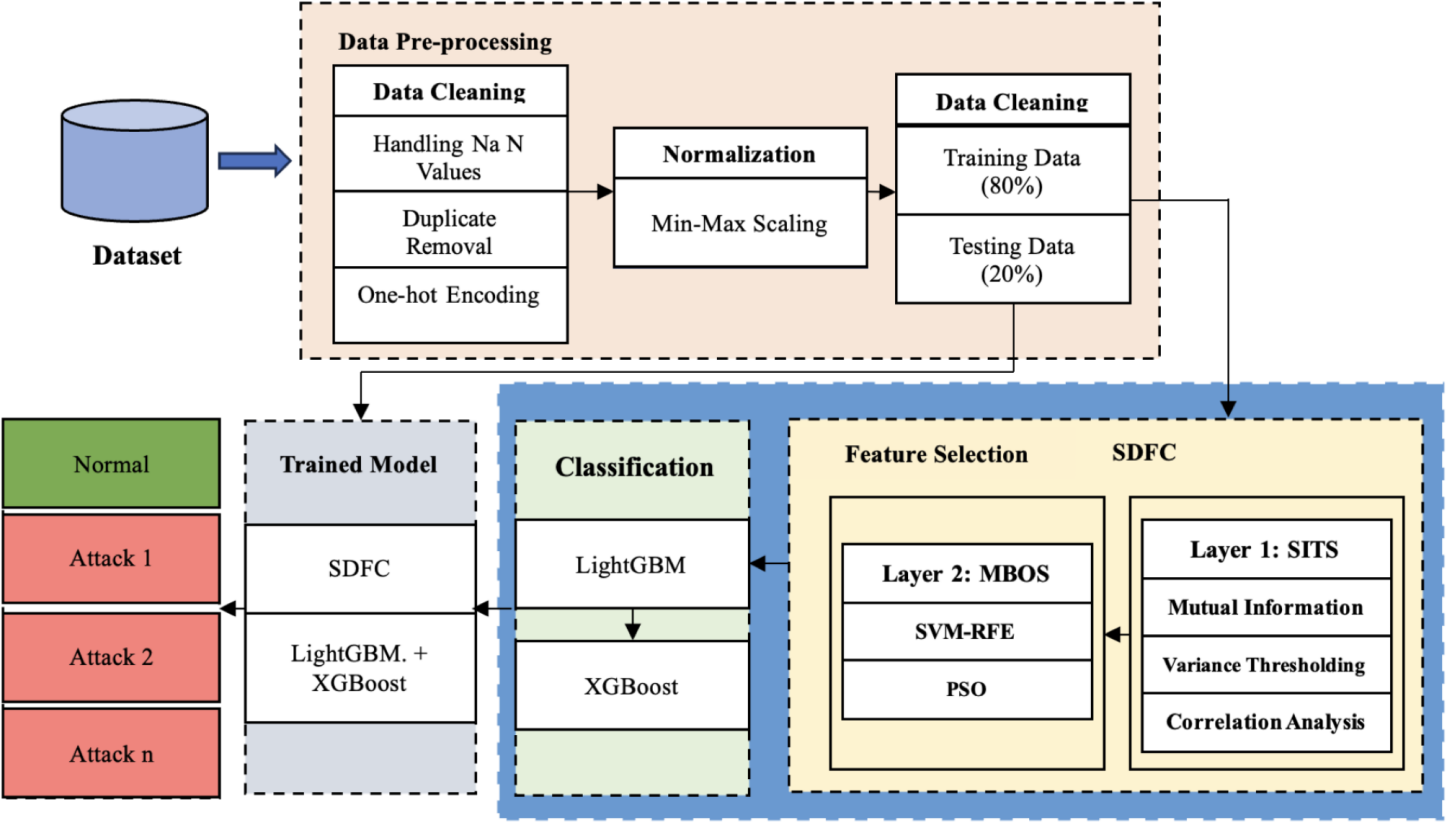
Proposed Intrusion Detection System.

The proposed IDS utilizes the TON-IoT network dataset, which integrates data from various sources, including IoT and IIoT sensors, network traffic, and operating systems, comprising over 22 million instances as shown in Fig. 1. The data preprocessing phase involves cleaning, which includes handling missing values through imputation and removing duplicates, and one-hot encoding for categorical features to convert them into a suitable numerical format. Normalization is performed using min-max scaling to standardize numerical features, ensuring they are on a comparable scale. In the proposed system the dataset is splited into training and testing to maintain equal class distribution by stratified sampling. In the feature selection subsystem, a SDFC approach is applied. The first layer, known as Statistical and Information-Theoretic Selection (SITS), employs the methods namely mutual information, variance thresholding, and correlation analysis to filter features. The second layer, Model-Based Optimization Selection (MBOS), further refines the features through techniques such as SVM-RFE and PSO. The proposed IDS operates in two stages: the first stage employs LightGBM to detect anomalies by classifying network traffic into normal or anomalous categories, while the second stage uses XGBoost to further categorize the detected anomalous traffic into specific attack types. This two-stage classifier approach enhances detection accuracy and efficiency, leveraging selected features to improve performance and reduce false alarms.

### Dataset

The TON-IoT network dataset serves as the basis for the proposed system. It encompasses 22,340,022 data instances and includes two target classes namely, one for categorizing data as normal or an attack, and second target class is employed to classify the attacks into ten distinct categories namely, normal and nine specific attacks which represented Table 2.

**Table 2:** Classes description in network TON_IoT.

Binary Classifications		Multi-class Classifications	
Type of classes	Instances	Type of classes	Instances
Normal	300,000	Normal	300,000
Attack	161,043	Backdoor	20,000
		DDoS	20,000
		DoS	20,000
		Injection	20,000
		Password	20,000
		Ransomware	20,000
		Scanning	20,000
		XSS	20,000
		MITM	1043

### Data Pre-processing

Data preprocessing is an essential subsystem for preparing the TON-IoT dataset for effective machine learning analysis. This subsystem ensures that the data is clean, standardized, and appropriately formatted to enhance the performance of subsequent machine learning models.

#### Data cleaning

The first step involves resolving issues of missing values and duplicates in the TON-IoT dataset. Missing values, which may arise from incomplete data collection or errors, are addressed through imputation. For numerical features, missing values are replaced with the mean value of that feature derived from the remaining data. For categorical features, imputation is done using the most frequent category. Additionally, any duplicate records are detected and removed to ensure that each data instance is unique, preventing potential skewing of results from over-represented entries. One-hot encoding is employed to transform categorical features into a numerical format suitable for machine learning models. For example, consider the “proto” feature, which includes different network protocols like “TCP,” “UDP,” and “ICMP.” One-hot encoding transforms this single feature into three binary columns: “proto_TCP,” “proto_UDP,” and “proto_ICMP.” Each data point is represented by a row where only the column corresponding to its protocol is marked with a 1, while the others are marked with 0. Similarly, the “service” feature, which might have categories such as “HTTP,” “FTP,” and “SSH,” is encoded into separate binary columns for each service type. Each data point will have a 1 in the column that matches its service type. For the “conn_state” feature, which denotes connection states like “ESTABLISHED,” “CLOSED,” and “SYN_SENT,” one-hot encoding creates individual binary columns for each state. This approach preserves the categorical information by representing each category with a distinct column and a binary value, allowing machine learning models to process the data effectively. Although one-hot encoding techniques increases the features and dimensionality, it avoids introducing any artificial ordinal relationships among categories, ensuring that the categorical variables are appropriately represented.

#### Normalization

Min-max scaling is essential for standardizing numerical features to ensure they are on a comparable scale, which improves the performance of machine learning models. Features like “byte_count,” “packet_size,” and “duration” can have widely varying ranges; for example, “byte_count” may range from 0 to 10,000, while “packet_size” might span from a few bytes to several kilobytes. Min-max scaling adjusts these features to a common range, typically [0, 1]. For instance, a “byte_count” value of 5,000 would be transformed using the minimum and maximum values of the feature to fit within this range. This process ensures that all numerical features contribute equally to the machine learning model, preventing features with larger scales from dominating the model’s training and enhancing its accuracy and performance in detecting anomalies or attacks. The formula for min-max normalization is shown in Eq. 1.

1 $$\:{X}_{norm}=\frac{X-{X}_{min}}{{X}_{max}-{X}_{min}}$$

where X represents the original value of the data point, $\:{X}_{norm}$ denotes the normalized value, $\:{X}_{min}$​ is the minimum value of the variable, and $\:{X}_{max}\:$is the maximum value of the variable in the dataset.

#### Data splitting

**Fig. 2:**
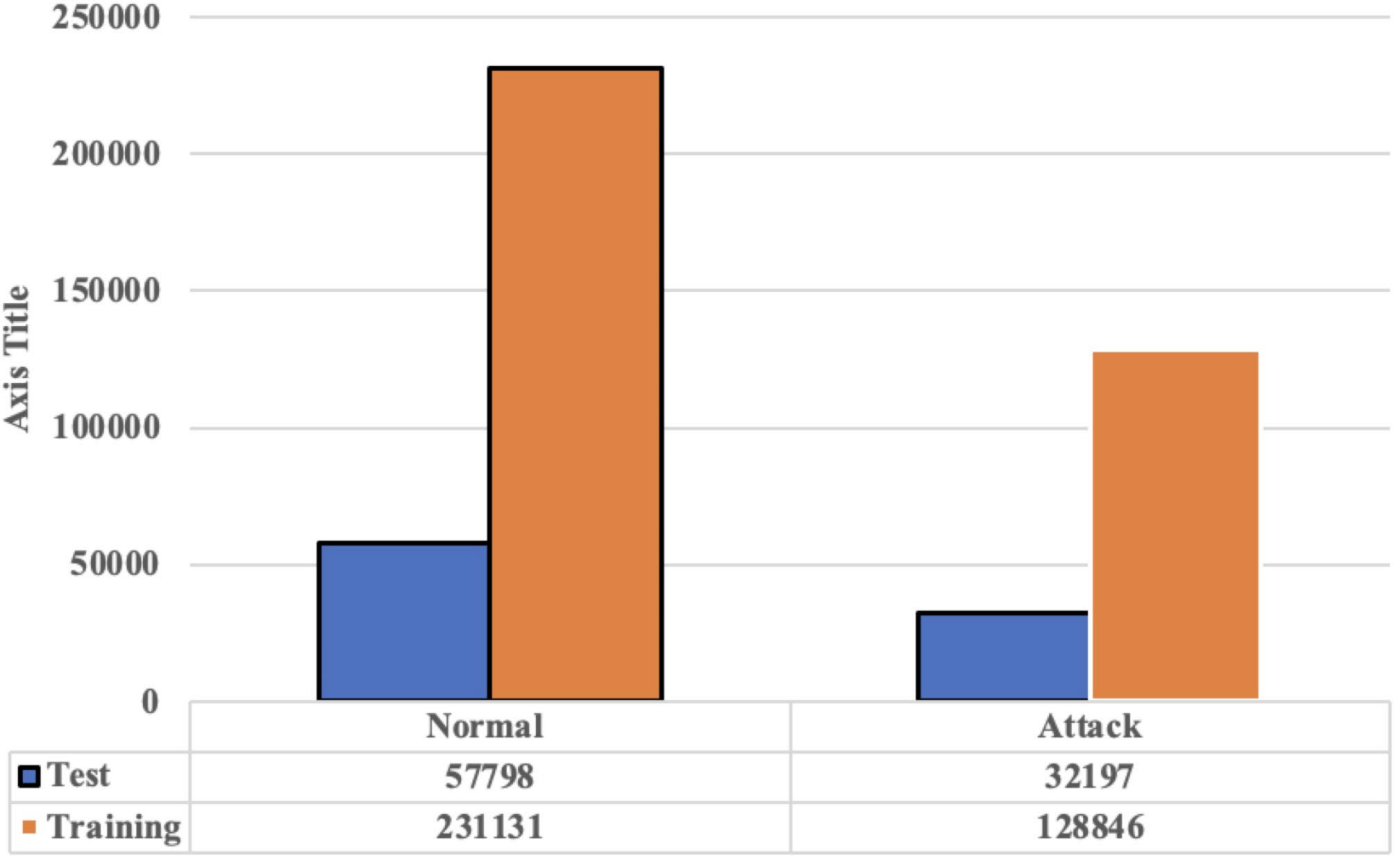
Distribution of instances for binary classification.

**Fig. 3:**
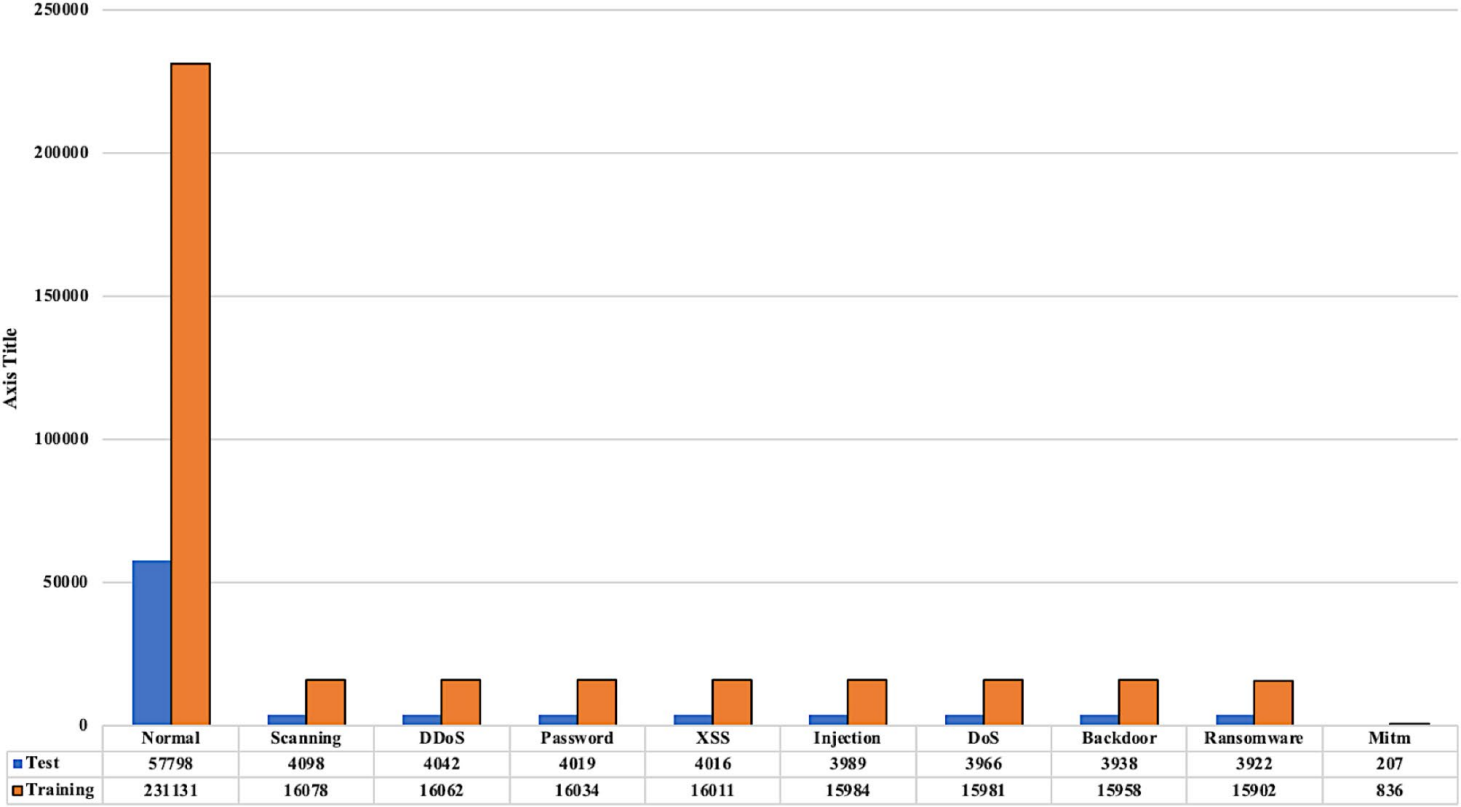
Distribution of instances for multi-class classification.

Data splitting involves partitioning the dataset into two main subsets: 80% is allocated to the training set, used for developing the model, while the remaining 20% is designated as the test set, used for evaluating the model’s performance. This method ensures that the model is effectively trained and its performance is assessed on new, unseen data. In the proposed system, stratified splitting technique is used to preserve the class distribution in both the training and test sets, accurately reflecting the class proportions in real-world IoT scenarios and ensuring a representative evaluation of the model. Figures 2 and 3 illustrate the distribution of instances for the binary and multi-class classifications.

## Feature selection method

The feature selection subsystem, a crucial part of the IDS, aims to identify and elect the relevant features from the dataset, thereby enhancing the performance and accuracy of the machine learning models. This subsystem uses a two-layer approach called SDFC, ensuring robust and efficient feature selection by leveraging diverse methodologies.

### Layer 1: Statistical and Information-Theoretic Selection (SITS)

In the proposed approach, layer 1 employs computation of three main functions namely Mutual Information, Variance Thresholding and correction analysis.

### Mutual information (MI)

The first step in Layer 1 involves calculating the MI between each feature and the target variable. MI measures the information obtained about an variable through another. For a feature X and the target variable Y, the MI *I(X; Y)* is defined in Eq. 2.

2 $$I\left(X;Y\right)=\sum\:_{x\in\:X}\sum\:_{y\in\:Y}p\left(x,y\right)\text{log}\:\left(\frac{p(x,y)}{p\left(x\right)p\left(y\right)}\right)$$

where $\:p\left(x,y\right)$ is the JPD of X and Y, and $\:p\left(x\right)$ and $\:p\left(y\right)$ are the PDF. Features with higher MI scores are retained, as they have a stronger dependency on the target variable.

#### Variance thresholding

Next, variance thresholding is applied to remove features with low variance, which are less informative for distinguishing between different classes. The variance $\:{\sigma\:}^{2}$ of a feature X is given in Eq. 3.

3 $${\:\sigma\:}^{2}=\:\frac{1}{N}{\sum\:}_{i=1}^{N}{({x}_{i}-\mu\:)}^{2}$$

where N is the number of samples, $\:{x}_{i}$ is the value of the feature for the i-th sample, and $\:\mu\:$ is the mean of the feature. Features with variance below a predefined threshold are eliminated.

#### Correlation analysis

To reduce redundancy, correlation analysis is performed to identify highly correlated features. The Pearson correlation coefficient r between two features X and Y is calculated in Eq. 4.

4 $$r\left(X,Y\right)=\frac{{\sum\:}_{i=1}^{N}({x}_{i}-{\mu\:}_{X}){(y}_{i}-{\mu\:}_{Y})}{\sqrt{{\sum\:}_{i=1}^{N}{({x}_{i}-{\mu\:}_{X})}^{2}{\sum\:}_{i=1}^{N}{({y}_{i}-{\mu\:}_{Y})}^{2}}}$$

where $\:{\mu\:}_{X}$​ and $\:{\mu\:}_{Y}$​ are the means of X and Y respectively. Features with a correlation coefficient r above a threshold are considered redundant, and only one feature from each group of correlated features is retained.

#### Layer 2: Model-Based optimization selection (MBOS)

In the proposed approach, model-based optimization selection approach is employed. The SVM-RFE and PSO is applied for efficient feature extraction.

#### Support vector Machine - Recursive feature elimination (SVM-RFE)

Layer 2 refines the feature selection using SVM-RFE, which recursively eliminates the least significant features. The importance of each feature is evaluated based on the weights w assigned by the SVM model. In each iteration, the feature with the smallest weight is removed. The ranking criterion for feature j is given in Eq. 5.

5 $${R}_{j}={w}_{j}^{2}$$

where $\:{w}_{j}$ is the weight of feature *j*. The process continues until the optimal number of features is selected.

#### Particle swarm optimization (PSO)

Finally, PSO is employed to further refine the feature subset. The position x and velocity v of a particle are updated and given in Eqs. 6 and 7.

6 $${v}_{i}\left(t+1\right)=\omega\:{v}_{i}\left(t\right)+{c}_{1}{r}_{1}\left({p}_{i}-{x}_{i}\left(t\right)\right)+{c}_{2}{r}_{2}\left(g-{x}_{i}\left(t\right)\right)$$

7 $${x}_{i}\left(t+1\right)={x}_{i}\left(t\right)+\:{v}_{i}(t+1)$$

where ω is the inertia weight, $\:{c}_{1}$​ and $\:{c}_{2}$ are cognitive and social coefficients, $\:{r}_{1}$and $\:{r}_{2}$ are random values, $\:{p}_{i}$is the particle’s best position, and $\:g$ is the global best position. PSO iteratively searches for the optimal feature subset that maximizes the model’s performance.

By combining the strengths of statistical, information-theoretic, and optimization-based methods, the SDFC algorithm ensures a robust and efficient feature selection process which improves the IDS accuracy as shown in algorithm 1.

**Figure:**
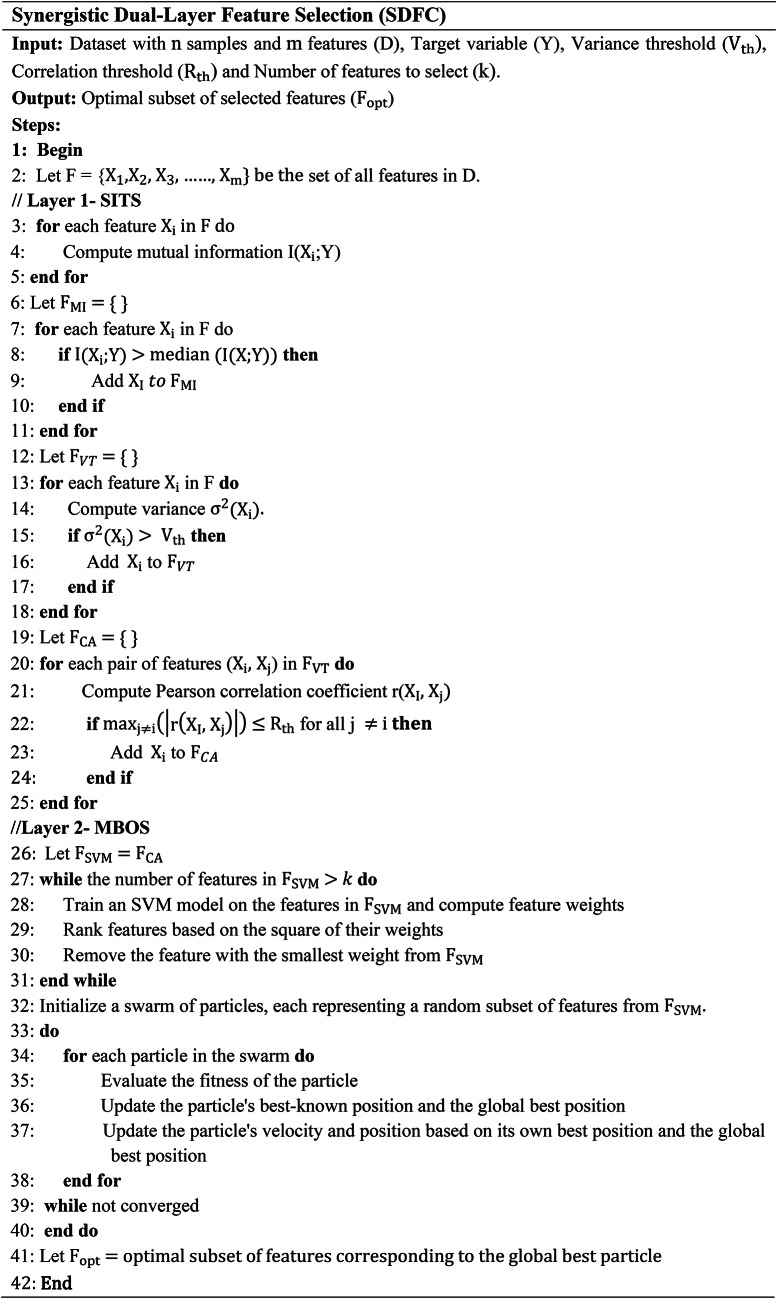
**Algorithm 1**

## Classification module

This paper presents a two-stage classifier for efficient classification of NIDS in the network. The proposed system employs LightGBM for anomaly detection in the first stage and XGBoost for misuse detection in the second stage. The system is evaluated using the TON-IoT dataset, with eight selected features. In the first stage, LightGBM processes the network traffic, distinguishing between normal and attack data. The attack traffic identified by LightGBM is then passed to XGBoost in the second stage, where it is further classified into various type of attacks. By utilizing the selected eight features, this two-stage classifier enhances computational efficiency, achieving improved accuracy and a lower false alarm rate in the IoT network.

In the first stage of the network IDS, LightGBM is utilized to detect anomalies in network traffic. The process begins with a dataset $\:D={\left\{\left({x}_{i},{y}_{i}\right)\right\}}_{i=1}^{N}$, where each instance $\:{x}_{i}\:\in\:{R}^{8\:\:}$represents an 8-dimensional feature vector. Each feature vector is associated with a label $\:{y}_{i}\:\in\:\left\{\text{0,1}\right\}\:$,where $\:{y}_{i}=0\:$denotes normal traffic and $\:{y}_{i}=1\:$denotes anomalous traffic.

The objective of LightGBM is to minimize the binary cross-entropy loss function, which measures the performance of the model in classifying traffic as normal or attack. The binary cross-entropy loss function is given in Eq. 8.

8 $$L\left({y}_{i},{\widehat{y}}_{i}\right)=\:-{y}_{i}\text{log}\left({\widehat{y}}_{i}\right)-\left(1-{y}_{i}\right)\:\text{l}\text{o}\text{g}(1-{\widehat{y}}_{i})$$

In Eq. 8, $\:{\widehat{y}}_{i}\:$represents predicted probability of occurrence for the *i*-th instance is anomalous. The loss function quantifies the error between the predicted probability $\:{\widehat{y}}_{i}\:$and the actual label $\:{y}_{i}$. A lower value of this loss indicates a better model performance.

To train the LightGBM model, the gradients $\:{g}_{i}\:$and hessians $\:{h}_{i}\:$ are computed for each instance. These are used to update the model parameters:

9 $${g}_{i}={\widehat{y}}_{i}-{y}_{i}$$

In Eq. 9, $\:{g}_{i}$ is the gradient, representing the difference between the predicted probability $\:{\widehat{y}}_{i}$ and the true label $\:{y}_{i}$. It indicates the direction in which the model needs to be adjusted to reduce the loss.

10 $$\:{h}_{i}={\widehat{y}}_{i}(1-{\widehat{y}}_{i})$$

In Eq. 10, $\:{h}_{i}\:$is the hessian, which is the second derivative of the loss function with respect to $\:{\widehat{y}}_{i}$. It measures the curvature of the loss function, providing insight into how the gradients should be adjusted.

The objective function for training each decision tree $\:{f}_{t}\left({x}_{i}\right)$in LightGBM is formulated as:

11 $$Objective=\:\sum\:_{i=1}^{N}\left({g}_{i}{f}_{t}\left({x}_{i}\right)+\frac{1}{2}{{h}_{i}{f}_{t}\left({x}_{i}\right)}^{2}\right)+\:{\Omega\:}{(f}_{t})$$

In Eq. (11), $\:{f}_{t}\left({x}_{i}\right)$ is the prediction made by the *t*-th decision tree for the *i*-th instance, $\:{\Omega\:}{(f}_{t})$ is the regularization term that penalizes the complexity of the tree to prevent overfitting.

The LightGBM model then classifies incoming network traffic into one of two categories: normal or anomalous. Traffic that is classified as anomalous by LightGBM is forwarded to the second stage for further classification. This two-stage approach improves the efficiency of the intrusion detection system by initially filtering out normal traffic and focusing resources on analyzing potentially harmful traffic in greater detail as shown in algorithm 2.

**Figure:**
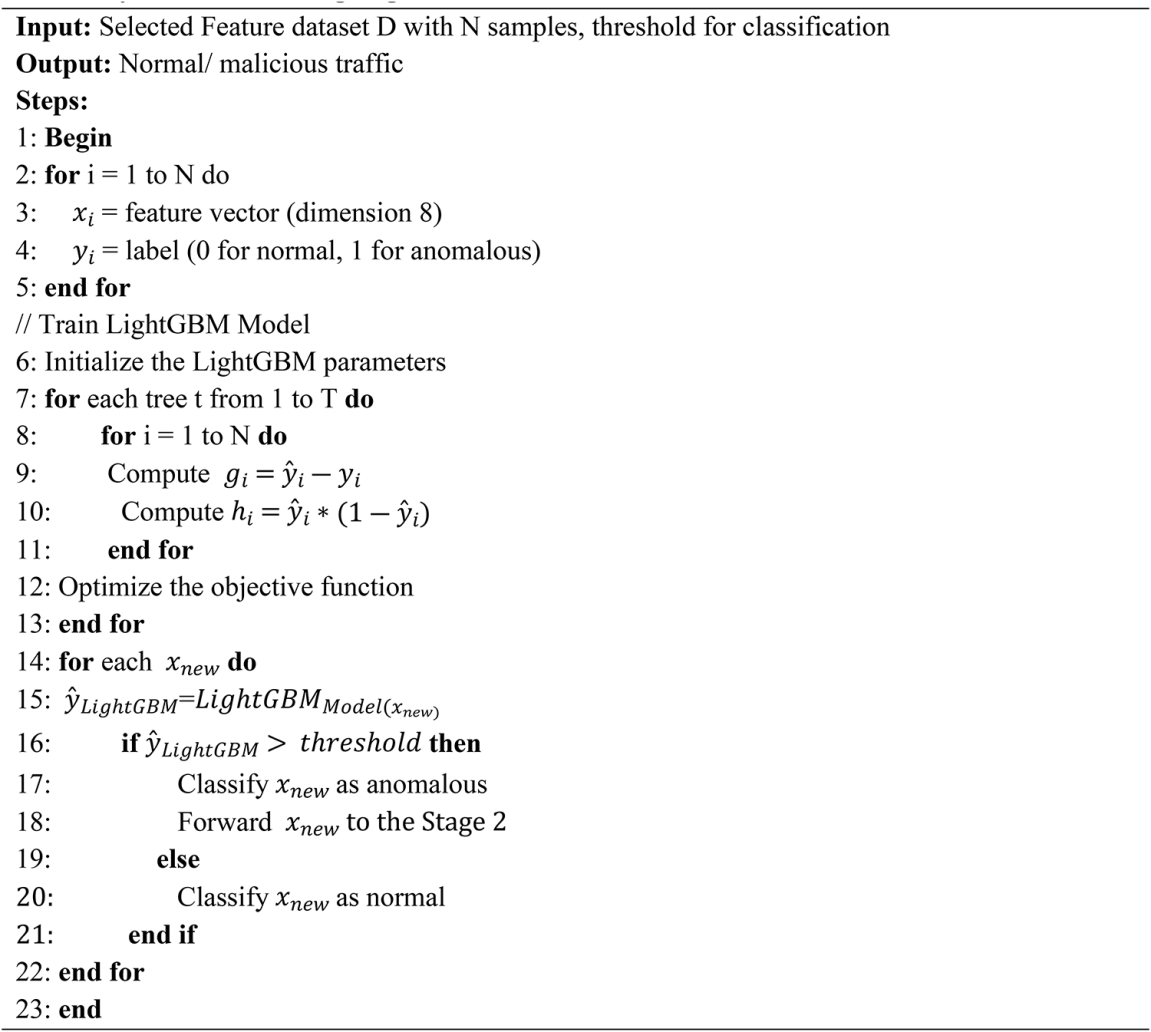
**Algorithm 2**

In the second stage of the network intrusion detection system, XGBoost is employed to classify anomalous traffic identified by LightGBM into specific attack categories. The dataset for this stage, denoted as $\:{D}_{attack}=\:{\left\{\left({x}_{i},{y}_{i}\right)\right\}}_{i=1}^{M}$, consists of feature vectors $\:{x}_{i}\in\:{R}^{8}$ and labels $\:{y}_{i}\in\:\left\{\text{1,2},\dots\:.K\right\},\:$where *K* represents the number of attack categories. XGBoost aims to minimize the multi-class cross-entropy loss function:

12 $$L\left({y}_{i},{\widehat{y}}_{ik}\right)=-\sum\:_{k=1}^{K}1\left({y}_{i}=k\right)\:\text{l}\text{o}\text{g}({\widehat{y}}_{ik})\:$$

In Eq. 12, $\:{\widehat{y}}_{ik}$ represents the predicted probability of the *i*-th instance belongs to class *k*, and $\:1\left({y}_{i}=k\right)$ is an indicator function that is 1 if $\:{y}_{i}=k$ and 0 otherwise. To optimize the model, XGBoost calculates gradients $\:{g}_{ik\:}$and hessians $\:{h}_{ik\:}$using Eqs. 13 and 14 for each class *k*:

13 $${g}_{ik}=\frac{\partial\:L({y}_{i},\:{\widehat{y}}_{ik})}{\partial\:{\widehat{y}}_{ik}}$$

14 $${h}_{ik}=\frac{{\partial\:}^{2}L({y}_{i},\:{\widehat{y}}_{ik})}{{\partial\:{\widehat{y}}_{ik}}^{2}}$$

The objective function for training each decision tree $\:{f}_{n}\left({x}_{i}\right)$ is computed using Eq. 15:

15 $$Objective=\:\sum\:_{i=1}^{M}({g}_{ik}{f}_{n}\left({x}_{i}\right)+\frac{1}{2}\:{{{h}_{ik}f}_{n}\left({x}_{i}\right)}^{2})+\:\lambda\:\bullet\:||\:{f}_{n} ||$$

where $\:\lambda\:$ is the regularization parameter to prevent overfitting, and ||$\:{f}_{n}$|| is the regularization term for tree complexity. The trained XGBoost model then classifies each instance of anomalous traffic into one of the predefined attack categories by selecting the class with the highest predicted probability using Eq. 16.

16 $$\widehat{y}_{XGBoost} = {\rm arg} \underbrace{max}_{k} \widehat{y}_{ik}$$

Thus, the second stage effectively categorizes the traffic identified as anomalous by LightGBM, providing a detailed classification of the specific types of attacks and enhancing the overall accuracy and specificity of the intrusion detection system. Algorithm 3 shows the misuse detection using XGBoost which classifies anomalous traffic data.

**Figure:**
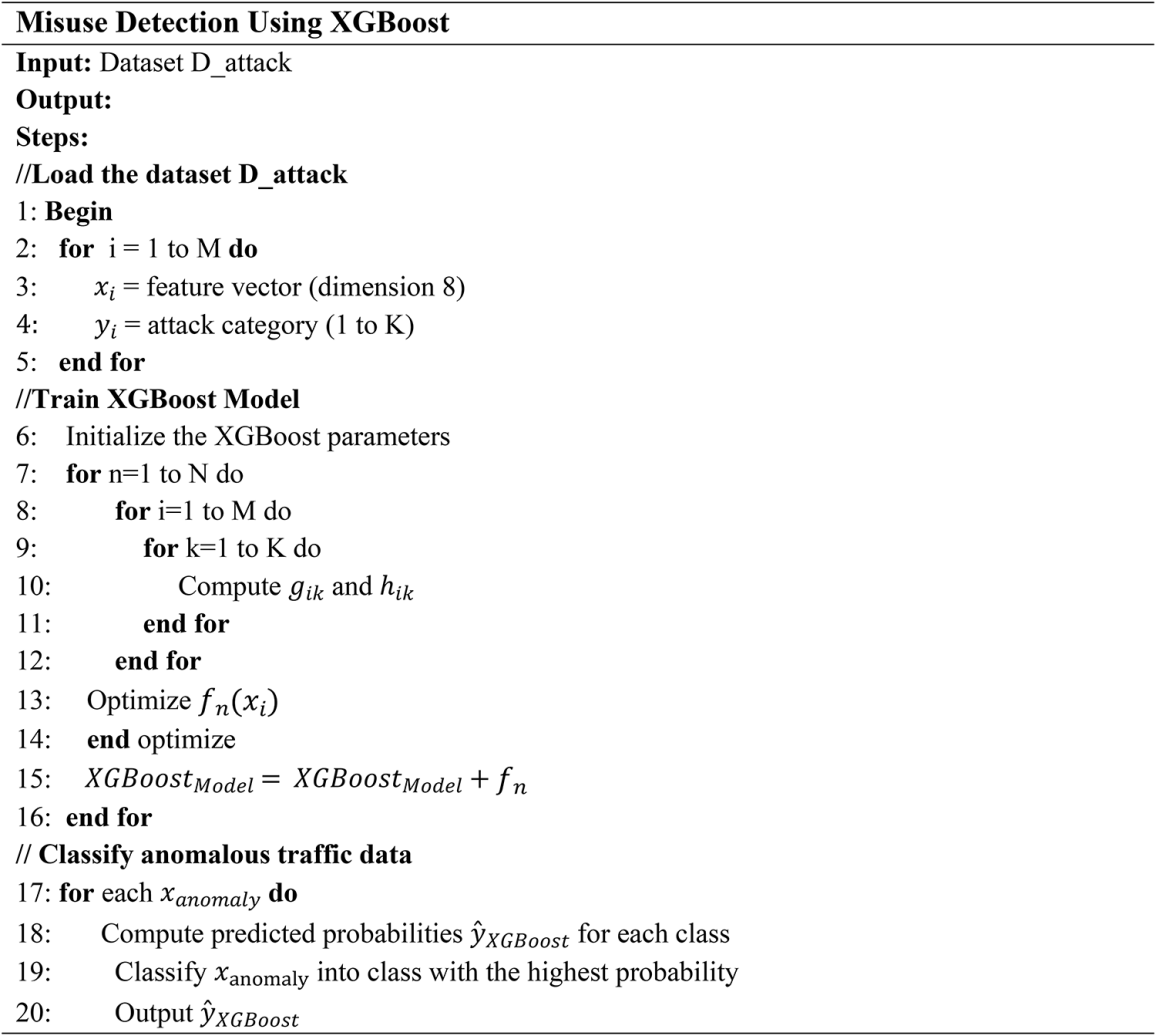
**Algorithm 3**

In the proposed work, LightGBM and XGBoost are selected for their exceptional efficiency, scalability, and precision. LightGBM is employed in the first stage due to its histogram-based learning approach, which effectively processes large-scale datasets like TON-IoT. It excels in handling high-dimensional data and addressing class imbalance while its leaf-wise tree growth strategy minimizes overfitting, ensuring reliable anomaly detection. In the second stage, XGBoost is utilized for its robustness and superior accuracy in multi-class classification. Its capability to manage diverse attack types, along with regularization techniques to prevent overfitting and insightful feature importance metrics, enhances both classification performance and interpretability. Together, these models form a highly scalable and precise two-stage intrusion detection system.

## Results and discussions

The experiments for the proposed work were conducted using Python (version 3.x) along with key libraries such as TensorFlow (version 2.x), Keras (version 2.x), Scikit-learn (version 0.24+), Pandas (version 1.x), NumPy (version 1.x), Matplotlib, and Seaborn. These libraries were selected for their efficient support for deep learning, data processing, and visualization tasks. The experiments were executed on a machine with an NVIDIA Tesla V100 GPU (16 GB memory, CUDA version 11.2), which provides the necessary computational power for deep learning model training. The machine was equipped with an Intel Xeon Gold 6248R CPU (24 cores, 3.0 GHz clock speed) to manage parallel tasks such as data preprocessing and model training. The system also included 64 GB of DDR4 RAM, ensuring smooth handling of large datasets and model training operations, while 1 TB SSD storage enabled fast data access and efficient saving of trained models. The operating system used was Ubuntu 20.04 LTS, providing a stable environment for running the required libraries and tools. This setup ensures that the computational requirements of the proposed deep learning models and adversarial attack simulations are met effectively, facilitating efficient experimentation and result reproduction. Table 3 summarizes the feature selection methods, including MI, Variance Thresholding, Correlation Analysis, SVM-RFE, PSO, and the proposed SDFC algorithm. Among various techniques, MI selects 20 features, Variance Thresholding selects 25, and Correlation Analysis narrows it down to 15 features. SVM-RFE and PSO further reduce the number to 10 and 12 features, respectively. The SDFC algorithm, which integrates multiple methods, is the most efficient, selecting only 8 features. This demonstrates its capability to efficiently retain the most relevant features while reducing the overall feature set for improved model performance and interpretability. The hyperparameters used for training the models, including LightGBM, XGBoost, and feature selection methods like SVM-RFE and PSO, are summarized in Table 3.

**Table 3:** Number of feature selected.

Feature Selection Method	Number of Features Selected
Mutual Information	20
Variance Thresholding	25
Correlation Analysis	15
SVM-RFE	10
PSO	12
Proposed SDFC	8

### Performance metrics

Performance metrics are important for evaluating and comparing the effectiveness of machine learning models^33,34^. The metrics used in this research are accuracy, precision, recall, and F1 score.

#### Accuracy

The proportion of correctly classified instances out of the total instances as shown in Eq. 17.

17 $$Accuracy=\:\frac{TP+TN}{TP+TN+FP+FN}$$

where, TP = True Positives (correctly detected attacks), TN = True Negatives (correctly identified normal instances), FP = False Positives (normal instances incorrectly identified as attacks) and FN = False Negatives (attacks that were not detected).

#### Precision

The ratio of true positive detections to the sum of true positives and false positives as shown in Eq. 18.

18 $$Precision=\:\frac{TP}{TP+FP}$$

#### Recall

The ratio of true positive detections to the sum of true positives and false negatives as shown in Eq. 19.

19 $$Recall=\:\frac{TP}{TP+FN}$$

#### F1 score

The harmonic mean of precision and recall, providing a single metric that balances both aspects as shown in Eq. 20.

20 $$F1\:Score=2\times\:\frac{Precision\:\times\:Recall}{Precision+Recall}$$

**Table 4:** Hyperparameters used for the proposed IDS.

Technique	Hyperparameter	Value
Data Preprocessing	One-Hot Encoding	No hyperparameters
	Min-Max Normalization	Range: [0, 1]
	Stratified Splitting	Training: 80%, Test: 20%
**Feature Selection (SDFC)**		
Layer 1: Statistical and Information-Theoretic Selection (SITS)	Mutual Information Threshold	Median MI score
	Variance Thresholding	0.01
	Pearson Correlation Coefficient	0.9
Layer 2: Model-Based Optimization Selection (MBOS)	SVM-RFE Kernel	Linear
	Regularization Parameter	1.0
	PSO Population Size	50
	PSO Iterations	100
	Inertia Weight	0.5
	Cognitive Coefficient	1.5
	Social Coefficient	2.0
**Machine Learning Models**		
LightGBM (Stage 1 - Binary Classification)	Boosting Type	Gradient Boosting
	Learning Rate	0.1
	Number of Leaves	31
	Max Depth	-1
	Number of Boosting Rounds	100
	Feature Fraction	0.8
	Bagging Fraction	0.8
	Bagging Frequency	5
XGBoost (Stage 2 - Multi-class Classification)	Booster	gbtree
	Learning Rate	0.1
	Max Depth	6
	Min Child Weight	1
	Subsample	0.8
	Colsample_bytree	0.8
	Number of Trees	100

Table 4 shows the hyperparameters used for the proposed system. The proposed system was evaluated by conducting a comparative analysis with several widely used classifiers. The results of the DT classifier with different feature selection methods are summarized in Table 5. The performance of the DT classifier with various feature selection methods highlights the effectiveness of the SDFC algorithm. Without feature selection, the DT classifier achieves an accuracy of 0.8720, with precision, recall, and F1 scores of 0.8680, 0.8750, and 0.8715, respectively. Among the feature selection methods, Mutual Information slightly improves these metrics, while variance thresholding shows a modest decrease. Correlation analysis yields better results than variance thresholding but falls short compared to advanced methods. SVM-RFE enhances performance, and PSO further improves metrics across the board. However, the SDFC algorithm outperforms all other methods, achieving the highest accuracy of 0.8890, and superior precision, recall, and F1 scores of 0.8860, 0.8925, and 0.8892, respectively. This demonstrates the SDFC algorithm’s superior ability to refine feature selection, leading to optimal classifier performance.

**Table 5:** Comparison of DT classifier with different feature selection methods.

Feature Selection Method	Accuracy	Precision	Recall	F1 Score
No Feature Selection	0.8720	0.8680	0.8750	0.8715
Mutual Information	0.8785	0.8735	0.8840	0.8787
Variance Thresholding	0.8690	0.8640	0.8745	0.8691
Correlation Analysis	0.8740	0.8685	0.8790	0.8737
SVM-RFE	0.8820	0.8780	0.8875	0.8826
PSO	0.8855	0.8820	0.8890	0.8854
Proposed SDFC	**0.8890**	**0.8860**	**0.8925**	**0.8892**

Table 6 presents a comparison of the RF classifier’s performance across various feature selection methods. The classifier demonstrates its highest performance with the Proposed SDFC method, achieving an accuracy of 0.9360, precision of 0.9320, recall of 0.9385, and an F1 score of 0.9352. This outperforms other feature selection methods, including PSO, SVM-RFE, and traditional approaches such as MI, variance thresholding, and correlation analysis. The results indicate that the Proposed SDFC method leads to superior classifier performance, emphasizing its effectiveness in selecting relevant features for the RF classifier.

**Table 6:** Comparison of RF classifier with different feature selection methods.

Feature Selection Method	Accuracy	Precision	Recall	F1 Score
No Feature Selection	0.9250	0.9210	0.9280	0.9245
Mutual Information	0.9280	0.9240	0.9300	0.9270
Variance Thresholding	0.9215	0.9180	0.9240	0.9210
Correlation Analysis	0.9245	0.9205	0.9280	0.9242
SVM-RFE	0.9310	0.9270	0.9340	0.9305
PSO	0.9335	0.9295	0.9360	0.9326
Proposed SDFC	0.9360	0.9320	0.9385	0.9352

Table 7 provides a comparison of the KNN classifier’s performance with other feature selection methods. The proposed SDFC method achieves the highest scores across all metrics, with an accuracy of 0.9180, precision of 0.9140, recall of 0.9210, and an F1 score of 0.9175. The enhanced performance of the proposed SDFC method highlights its effectiveness in improving the KNN classifier’s accuracy and overall performance by selecting the most relevant features.

**Table 7:** Comparison of KNN classifier with different feature selection methods.

Feature Selection Method	Accuracy	Precision	Recall	F1 Score
No Feature Selection	0.9000	0.8950	0.9050	0.9000
Mutual Information	0.9075	0.9030	0.9120	0.9074
Variance Thresholding	0.8950	0.8885	0.9040	0.8962
Correlation Analysis	0.9035	0.8980	0.9100	0.9040
SVM-RFE	0.9120	0.9075	0.9150	0.9111
PSO	0.9150	0.9110	0.9180	0.9145
Proposed SDFC	0.9180	0.9140	0.9210	0.9175

**Table 8:** Comparison of MLP classifier with different feature selection methods.

Feature Selection Method	Accuracy	Precision	Recall	F1 Score
No Feature Selection	0.9310	0.9300	0.9300	0.9300
Mutual Information	0.9342	0.9335	0.9340	0.9337
Variance Thresholding	0.9275	0.9268	0.9270	0.9269
Correlation Analysis	0.9321	0.9310	0.9320	0.9315
SVM-RFE	0.9360	0.9345	0.9365	0.9355
PSO	0.9378	0.9362	0.9380	0.9371
Proposed SDFC	0.9395	0.9380	0.9400	0.9390

Table 8 illustrates the performance of the MLP classifier with various feature selection methods. The proposed SDFC method demonstrates better performance in terms of accuracy (0.9395), precision (0.9380), recall (0.9400), and F1 score (0.9390). The proposed SDFC’s ability to enhance the MLP classifier’s metrics underscores its effectiveness in feature selection, leading to improved model performance.

**Table 9:** Comparison of LightGBM and XGBoost with different feature selection methods.

Feature Selection Method	Accuracy	Precision	Recall	F1 Score
No Feature Selection	0.9285	0.9278	0.9280	0.9279
Mutual Information	0.9317	0.9310	0.9315	0.9312
Variance Thresholding	0.9253	0.9245	0.9250	0.9247
Correlation Analysis	0.9308	0.9300	0.9305	0.9303
SVM-RFE	0.9335	0.9328	0.9340	0.9334
PSO	0.9352	0.9340	0.9355	0.9347
Proposed SDFC	0.9370	0.9365	0.9378	0.9371

Table 9 shows the comparison of LightGBM and XGBoost classifiers using different feature selection methods. The **Proposed SDFC** method demonstrates the best performance across all evaluated metrics, achieving the better results in accuracy (0.9370), precision (0.9365), recall (0.9378), and F1 score (0.9371). This suggests that the proposed system, with its optimized feature selection approach, significantly enhances model performance compared to the other existing methods, including Mutual Information, Variance Thresholding, Correlation Analysis, SVM-RFE, and PSO. The improvement in these metrics highlights the effectiveness of the SDFC method in selecting the most relevant features, reducing noise, and ultimately leading to more precise and reliable predictions for intrusion detection.

**Fig. 4:**
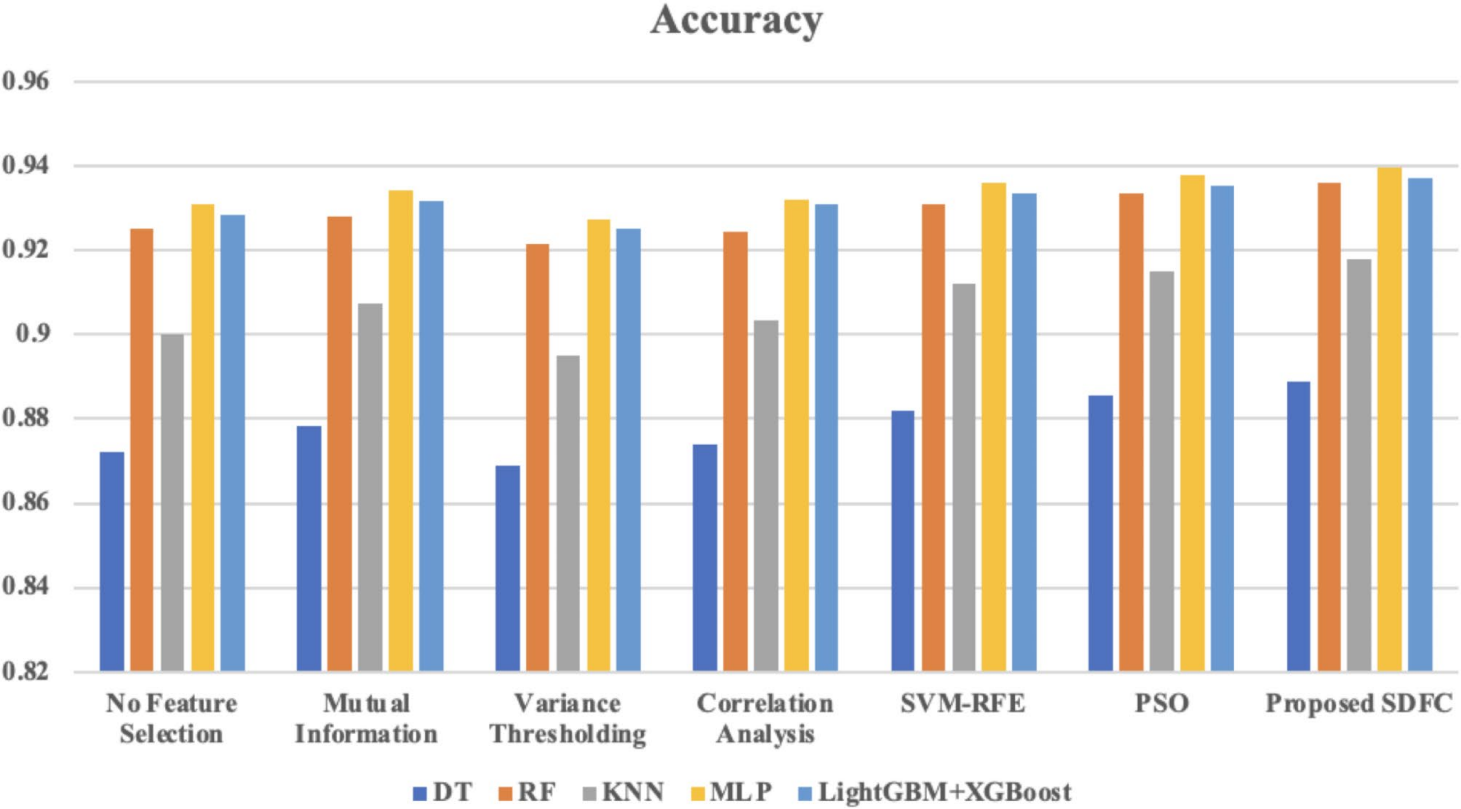
Comparison of accuracy across different classifiers using various feature selection method.

Figure 4 presents a comparison of accuracy across different classifiers using various feature selection methods. The accuracy of the models improves across all feature selection techniques when compared to the “No Feature Selection” baseline. Among the feature selection methods, Particle Swarm Optimization (PSO) and Support Vector Machine Recursive Feature Elimination (SVM-RFE) show notable performance improvements, with PSO achieving the highest accuracy in most cases. However, the proposed SDFC (Specialized Dynamic Feature Combination) method consistently yields the best results across all models, with accuracy scores surpassing those of other techniques. For instance, the SDFC method achieves an accuracy of 0.889 for DT, 0.936 for RF, 0.918 for KNN, 0.9395 for MLP, and 0.937 for LightGBM + XGBoost, highlighting its superiority in enhancing model performance.

**Fig. 5:**
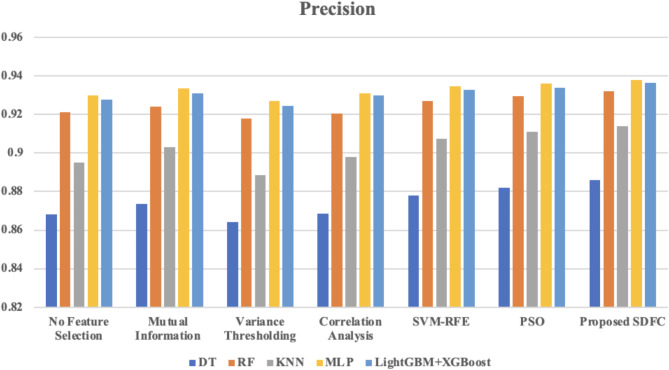
Comparison of precision across different classifiers using various feature selection method.

The precision values across various classifiers and feature selection methods are depicted in Fig. 5. The “No Feature Selection” approach yields relatively high precision, but applying feature selection generally enhances performance. Mutual Information and Correlation Analysis provide moderate gains, while SVM-RFE and PSO show further improvements, especially with classifiers like MLP and LightGBM + XGBoost. The proposed Synergistic Dual-Layer Feature Selection (SDFC) method consistently achieves the highest precision across all classifiers, reaching 0.938 with MLP and 0.9365 with LightGBM + XGBoost. These results emphasize the SDFC method’s superior ability to improve precision in identifying malicious activities in IoT networks.

**Fig. 6:**
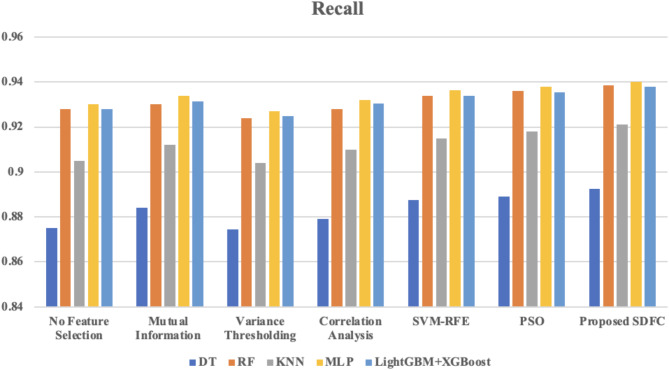
Comparison of recall across different classifiers using various feature selection method.

The recall results presented in Fig. 6 illustrate the effectiveness of different feature selection methods across multiple classifiers for intrusion detection. Without feature selection, recall scores are relatively high but show improvement when feature selection methods are applied. Mutual Information and Correlation Analysis yield moderate recall enhancements, while SVM-RFE and PSO provide further improvements, especially with classifiers like MLP and LightGBM + XGBoost. The proposed Synergistic Dual-Layer Feature Selection (SDFC) method consistently achieves the highest recall across all classifiers, with notable results such as 0.94 for MLP and 0.9378 for LightGBM + XGBoost. These findings underscore the SDFC method’s superior ability to enhance recall in detecting malicious activities in IoT networks.

**Fig. 7:**
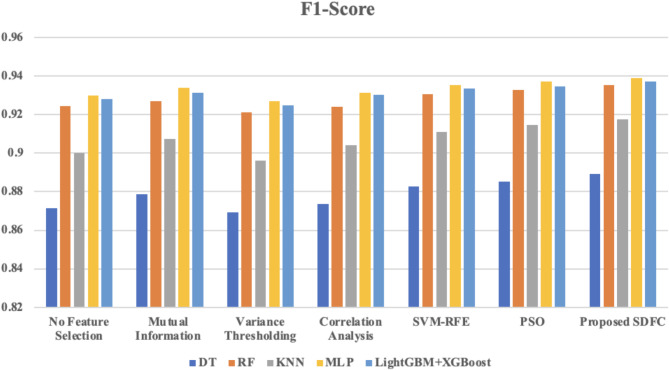
Comparison of F1-score across different classifiers using various feature selection method.

Figure 7 presents the F1 score results across various classifiers, showcasing the impact of different feature selection methods on intrusion detection performance. Without feature selection, the F1 scores are relatively high, but applying feature selection generally improves these results. Mutual Information and Correlation Analysis provide moderate F1 score enhancements, while SVM-RFE and PSO yield further improvements, particularly with classifiers such as MLP and LightGBM + XGBoost. The proposed Synergistic Dual-Layer Feature Selection (SDFC) method consistently achieves the highest F1 scores across all classifiers, with values like 0.939 for MLP and 0.9371 for LightGBM + XGBoost. These results underscore the SDFC method’s superior ability to enhance the F1 score in detecting malicious activities within IoT networks, reflecting a balance between precision and recall.

**Table 10:** Comparison of training and testing time.

Model	Training Time (s)	Testing Time (s)
DT	24.5678	0.0986
RF	52.3412	0.1765
KNN	36.8901	0.1648
MLP	50.2123	0.1658
XGBoost + LightGBM	58.1237	0.1895

**Fig. 8:**
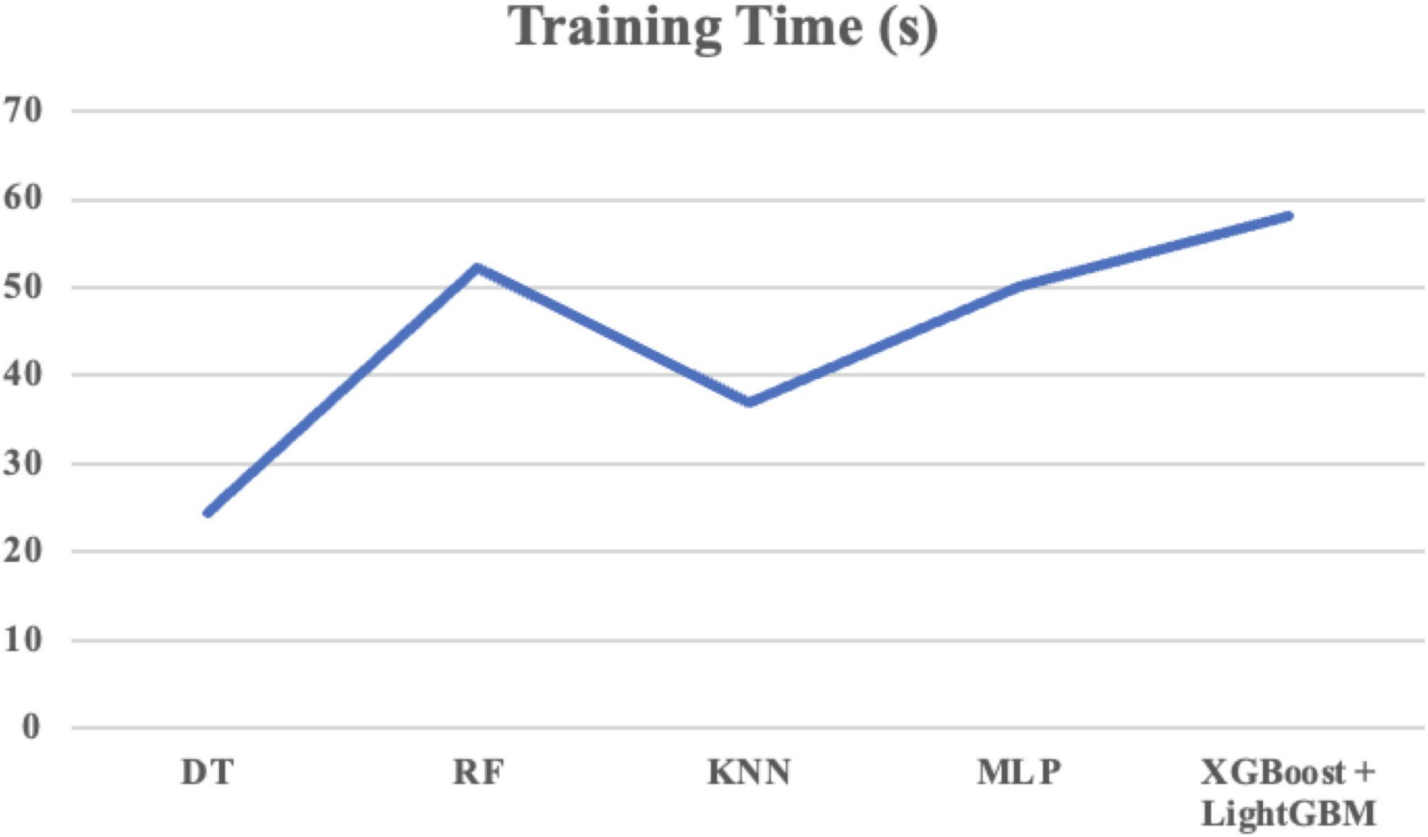
Training Time for various classifiers.

**Fig. 9:**
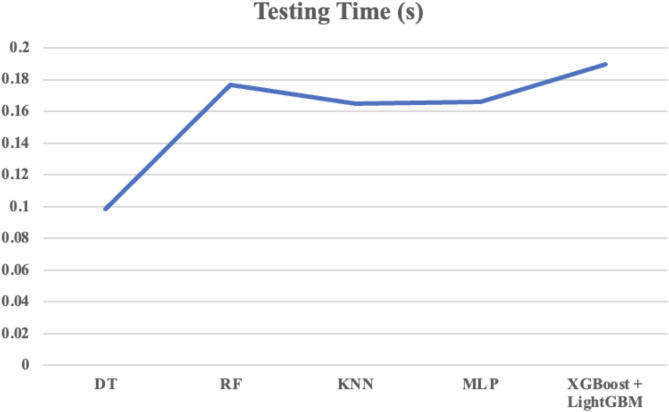
Testing Time for various classifiers.

Table 10 compares the training and testing times of various machine learning models. The combination of XGBoost + LightGBM offers superior performance despite longer training times (58.12 s) compared to simpler models like DT, RF, KNN, and MLP. This is due to the complexity of the boosting algorithms used, which build multiple decision trees to enhance accuracy. However, both models are optimized for fast predictions, with a testing time of 0.1895 s, making them suitable for real-time applications. XGBoost and LightGBM excel in handling large datasets and capturing complex patterns, offering scalability, regularization, and robustness that simpler models lack. Thus, the trade-off in training time is justified by their improved accuracy, efficiency, and scalability, making them the best choice for demanding tasks. The trade-off between training and testing time for the classifiers is visually represented in Figs. 8 and 9.

## Conclusions and future works

The proposed IDPS offers a comprehensive solution to enhance security in IoT networks. By incorporating a multi-faceted approach, including data pre-processing, advanced feature selection, diverse ML models, and proactive threat prevention, the system effectively addresses the unique security challenges posed by IoT environments. The data pre-processing subsystem plays a crucial role in ensuring data quality and consistency, which is essential for accurate model training and evaluation. The SDFC algorithm further enhances the system’s performance by combining statistical, information-theoretic, and optimization-based methods to refine feature relevance. The classification subsystem uses a two-stage classifier, LightGBM and XGBoost, to efficiently categorize network traffic as normal or malicious. Experimental results using the TON-IoT dataset demonstrate that the proposed IDS outperforms traditional methods, achieving higher accuracy (0.9370), precision (0.9365), recall (0.9378), and F1 score (0.9371). These metrics highlight the effectiveness of the IDS in accurately detecting malicious activities within IoT networks. Future research on the proposed IDS could focus on several key areas. First, exploring advanced feature engineering and domain-specific techniques might enhance feature selection and model performance. Second, implementing adaptive learning to continuously update models based on new data and emerging threats could improve resilience against evolving attacks. Third, integrating the IDS with additional security measures, such as firewalls and anomaly detection systems, could offer a more comprehensive security framework. Fourth, optimizing the system for scalability and real-time processing will be crucial for practical use in large-scale IoT deployments. Fifth, further testing with real-world IoT data will validate its effectiveness and adaptability. Lastly, enhancing user interfaces and automated response mechanisms could improve overall efficiency and usability. Addressing these areas will lead to more robust and adaptive intrusion detection and prevention systems for IoT networks.

## Data Availability

All data generated or analyzed during this study are included in this published article.
